# Adverse childhood experiences among high-risk children living in socially vulnerable areas

**DOI:** 10.1590/0034-7167-2024-0247

**Published:** 2025-03-17

**Authors:** Leticia Gramazio Soares, Sabrina dos Santos Tomé, Isabella Schroeder Abreu, Maicon Henrique Lentsck, Tatiane Baratieri, Jorge Marcelo Sauka, Isadora Bussolaro Viana, Kelly Cristina Michalczyszyn

**Affiliations:** I Universidade Estadual do Centro-Oeste Guarapuava Paraná Brazil Universidade Estadual do Centro-Oeste. Guarapuava, Paraná, Brazil; II Centro Universitário Campo Real Guarapuava Paraná Brazil Centro Universitário Campo Real. Guarapuava, Paraná, Brazil

**Keywords:** Adverse Childhood Experiences, Comprehensive Health Care, Vulnerable Populations, Child Development, Child, Experiencias Adversas de la Infancia, Atención Integral de Salud, Poblaciones Vulnerables, Desarrollo Infantil, Niño

## Abstract

**Objectives:**

to identify the occurrence of adverse childhood experiences (ACEs) among children classified as high-risk at birth.

**Methods:**

this quantitative, cross-sectional, and descriptive study was conducted within an Intermunicipal Health Consortium in Paraná from September 2022 to February 2023, involving 45 caregivers of high-risk children. Data collection took place at the participants’ homes using three questionnaires. The results were analyzed descriptively, based on the theory of the adverse childhood events tree.

**Results:**

the prevalence of adverse childhood events was 18.6%. Regarding the types of events, 64.3% reported violence; 28% reported parental divorce; 22.2% reported substance abuse by caregivers; 73.3% experienced difficulty acquiring basic necessities; 62.2% were unemployed and/or had low income; 55.6% lived in conflict-prone areas; and 44.4% lacked access to sewage systems.

**Conclusions:**

adverse childhood events are multifactorial and cross-sectoral, posing significant threats to child development. The 2030 Agenda proposes dimensions for addressing this issue by investing in childhood.

## RESUMEN

**Objetivos:**

identificar la ocurrencia de experiencias adversas en la infancia entre niños clasificados como de alto riesgo al nacer.

**Métodos:**

estudio cuantitativo, transversal y descriptivo, realizado en un Consorcio Intermunicipal de Salud en Paraná, de septiembre de 2022 a febrero de 2023, con 45 cuidadores de niños de alto riesgo. La recolección de datos se llevó a cabo en los domicilios de los participantes mediante la aplicación de tres cuestionarios. Los resultados fueron analizados de manera descriptiva, basados en la teoría del árbol de eventos adversos en la infancia.

**Resultados:**

la prevalencia de eventos adversos en la infancia fue del 18,6%. En cuanto a los tipos de eventos, el 64,3% reportó violencia; el 28% reportó divorcio parental; el 22,2% reportó abuso de sustancias por parte de los cuidadores; el 73,3% experimentó dificultades para adquirir productos básicos; el 62,2% se encontraba en situación de desempleo y/o bajos ingresos; el 55,6% residía en zonas de conflicto; y el 44,4% no tenía acceso al sistema de alcantarillado.

**Conclusiones:**

los eventos adversos en la infancia son multifactoriales e intersectoriales, representando amenazas significativas para el desarrollo infantil. La Agenda 2030 propone dimensiones para abordar esta problemática mediante la inversión en la infancia.

## INTRODUCTION

Early childhood, encompassing the first six years of life, represents a critical stage in an individual’s development when cerebral architecture is established, characterized by rapid neuroplasticity^(1)^. During this period, the brain is shaped by every experience the child undergoes^(2)^, marking a phase in which they acquire the capacity to learn^(3)^. The manner in which learning is stimulated during this period lays the foundation for human development in subsequent years^(4)^.

When a child faces negative situations, these experiences can cause physical and/or psychological harm, potentially interfering with brain development, deregulating the immune and neuroendocrine systems, impairing learning, hindering the formation of bonds, and impacting social relationships in the short, medium, and long term. The literature defines such situations as adverse childhood experiences (ACEs)^(4,5)^.

Children exposed to ACEs are at a higher risk of substance use/abuse, unintended pregnancies, chronic diseases, sexually transmitted infections, and psychopathologies upon reaching adulthood, in addition to experiencing difficulties in achieving their intellectual, social, and economic potential^(4,5,6)^.

With the introduction of the Sustainable Development Goals (SDGs), the focus in child health has shifted toward improving outcomes in child development alongside reducing mortality^(1,7)^. As a result, childhood has become an international priority, with the aim of providing all children with the opportunity to reach their full developmental potential^(5)^, particularly those who are underprivileged, to ensure a healthier planet for present and future generations^(8)^.

As such, all 193 United Nations (UN) Member States, including Brazil, have committed to adopting and implementing an agenda that fulfills the 17 SDGs, representing a global action plan to enhance societal quality of life by 2030^(8)^.

The Early Childhood Development Action Network, which comprises the United Nations Children’s Fund, the World Bank, and the World Health Organization, has proposed a care model encompassing health, nutrition, responsive caregiving, protection, safety, and learning from the earliest stages of life^(9)^. In Brazil, strengthening this agenda led to the creation of the Legal Framework for Early Childhood, which sets forth principles and guidelines for public policies during the early years of life^(10)^.

However, the classification of children as high-risk at birth has become increasingly prevalent in recent years, reflecting growing awareness among health services of the negative experiences that may hinder children’s growth and development^(11)^. A study on hospitalization during early childhood indicates that such conditions can negatively impact growth and development both in the short and long term^(12)^.

Social vulnerability is recognized in the literature as an ACE because it exposes children to the consequences of social inequality, including poverty, social exclusion, and limited access to education, employment, healthcare, recreation, food, and culture^(13,14)^. Studies suggest that the most effective way to invest in childhood is to reduce inequalities and build a society with sustainable living conditions^(15,16)^.

Evidence from neuroscience underscores the urgent need to expand multisectoral coverage during childhood, incorporating health, nutrition, protection against violence, and education to ensure children develop the necessary skills to become healthy and productive adults^(15)^.

In this context, it is critical to promote social and governmental awareness regarding ACEs and their impact on human development. Therefore, the topic must be explored, monitored, and addressed by public authorities through strategies for prevention and intervention. This represents a gap identified in the existing literature^(17,18)^ that this study aims to address by bringing the issue to the forefront of discussion and dissemination within the scientific community. Now is the time to broaden the dialogue to support managers and health professionals in recognizing early childhood as a window of opportunity for human development. Actions focused on childhood should integrate the SDGs into health policies, with the goal of preventing and reducing exposure to ACEs.

## OBJECTIVES

To identify the occurrence of ACE among children classified as high-risk at birth.

## METHODS

### Ethical Aspects

This study adhered to the guidelines of Resolution No. 466/2012 of the National Health Council and was approved by the Research Ethics Committee for Human Subjects. All research participants signed an Informed Consent Form (ICF).

### Study Design, Period, and Location

This quantitative, cross-sectional, and descriptive study was conducted through home visits to families whose high-risk children were under follow-up care at the Pediatric Outpatient Clinic, a service provided by the Intermunicipal Health Consortium of Guarapuava and Pinhão (CIS-GAP), located in Guarapuava, Paraná, Brazil. The purpose of this service is to offer consultations, exams, and specialized procedures to the populations of the participating municipalities. The choice of this institution was based on its role in assisting newborns classified as high-risk—those with a greater likelihood of adverse outcomes related to mortality and morbidity. The study was conducted from September 2022 to February 2023. The Strengthening the Reporting of Observational Studies in Epidemiology (STROBE) guidelines were followed during the study’s development.

### Study Population; Inclusion and Exclusion Criteria

The study population consisted of caregivers of children classified as high-risk at birth who lived in socially vulnerable areas. Inclusion criteria for participants were: caregivers with legal guardianship of high-risk children at birth who were receiving follow-up care at the CIS-GAP outpatient clinic between September 2022 and February 2023; children aged between zero and two years; residency in peripheral neighborhoods of Guarapuava characterized by social vulnerability; and the availability of the caregiver, who had to be of legal age, to respond to the questionnaire. The socially vulnerable areas included in the research were recognized by the municipal health service and were characterized by low income, unemployment, infrastructure issues, and environmental precariousness.

Exclusion criteria were: caregivers with cognitive impairments preventing them from responding; caregivers who could not be found at home on the day of the visit; or a change of address.

Initially, a survey of high-risk children receiving services was conducted, totaling 242 children. After applying the inclusion criteria, 92 children were selected to participate in the study, of which 45 effectively participated. Losses occurred for the following reasons: change of address (26); caregiver not found at home (18); refusal to participate (3).

Data collection was conducted in the families’ homes due to the inability to perform it at the service location, as it was undergoing physical restructuring during the collection period. Three structured questionnaires were administered: the first contained sociodemographic data; the second addressed the child’s health history, access to services, nutrition, growth/development, and support network; and the third included specific questions about ACEs. The questionnaires were administered by researchers who had been trained and qualified for the task.

### Analysis of Results and Statistics

The results were analyzed descriptively, with absolute and relative frequencies presented. To illustrate the findings obtained in the research, the “The Pair of Aces”^(19)^ theory, or the ACE tree, was used as a reference. This theory is graphically represented as a tree, with the leaves symbolizing the consequences of growing up in a context marked by poverty and inequality.

## RESULTS

A total of 45 caregivers of high-risk children at birth participated in the study, of whom 17 (37.8%) were between 20 and 25 years old; 29 (64.4%) lived with partners, while 16 (35.5%) did not; 29 (64.4%) identified as white, and 16 (35.5%) as mixed-race (pardos); 29 (64.4%) professed the Catholic faith, seven (15.6%) were Evangelical, and nine (20%) reported having no religion; 17 (37.8%) had not completed high school, and 11 (24.4%) had completed high school; one caregiver (2.2%) had started a college degree, and two (4.4%) had completed higher education. Additionally, one participant had no access to formal education.

The children’s ages ranged from one to 24 months, with 15 (33.3%) corresponding to the age group of 12 to 18 months. Regarding the conditions that determined their high-risk classification at birth, 18 (40%) were premature; 10 (22.2%) had low birth weight; eight (17.8%) had congenital malformations; and nine (20%) had vertically transmitted diseases.

Regarding breastfeeding, the only child under six months included in the study was exclusively breastfed; 55.6% of the children were receiving, in addition to breast milk and/or formula, complementary foods. Among these, 93.3% consumed fruits and vegetables; 91.1% consumed greens; 95.6% ate meat; 97.8% consumed rice, cassava, potatoes, flour, and pasta; 62.2% consumed processed meats; 66.7% drank sugary beverages; and 71.1% consumed processed foods such as filled cookies and snacks.

Eight caregivers did not have the child’s health card, making it impossible to collect information on growth and development. However, among the 37 available health cards, the following data were observed: 22.2% had delayed neuropsychomotor development; 66.7% were diagnosed with a chronic illness; 28.9% had been hospitalized after 28 days of life; and 13.3% were taking daily medication.

The findings regarding ACEs identified in this research were presented based on an adaptation of the image originally titled “The Pair of Aces”^(19)^, emphasizing the importance of safe environments for childhood.

**Figure 1:**
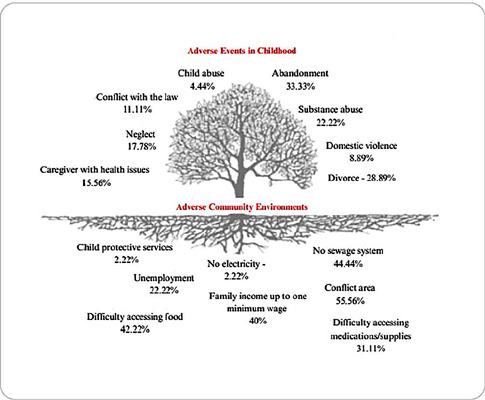
Tree of Adverse Childhood Experiences

The analysis of the results through the graphical representation of the tree allows for the systematic categorization of ACEs, organizing the data into two classes. The first represents individual situations—specifically the ACEs themselves—symbolized by branches and leaves and refers to the adversities experienced by the children who participated in the study. The second class pertains to environmental situations, represented by the soil, termed the adverse community environment (Figure 1).

The prevalence of ACEs in the general population was 18.6%. Regarding the occurrence and types of ACEs experienced by the 45 children included in the study, the findings were as follows: 64.3% of families reported situations of violence, including neglect, abandonment, and domestic violence; 28.8% of families experienced parental divorce; 22.2% reported substance abuse by the primary caregivers; 15.6% of caregivers had health issues that interfered with child care; and 11.1% of caregivers reported having conflicts with the law.

With respect to the environments to which the children were exposed, there was significant difficulty for families in acquiring food, medicine, and other essential supplies, as reported by 73.3% of families; 62.2% reported unemployment and low income; 55.6% lived in conflict-prone areas; 44.4% did not have access to a sewage system; 2.2% did not have access to an electricity network; and 2.2% were dealing with issues involving the Child Protection Council.

ACEs can encompass various types of situations, as noted. In terms of the number of ACEs each child was exposed to, the distribution was as follows: 20 children (44.5%) were exposed to two to five events; 11 children (24.5%) to six to nine events; 10 children (22.2%) to only one event; and four children (8.8%) were exposed to more than 10 events, illustrating the coexistence of multiple adverse events within the same child.

## DISCUSSION

Reducing exposure to ACEs is a global challenge, as social and structural issues emerge within the study context and drive the discussion toward the needs of child development. The literature indicates a direct relationship between the number of ACEs and developmental difficulties in children: the greater the number of adverse experiences a child is exposed to, the more significant the consequences^(20)^. The results of this study reinforce this relationship, demonstrating that most children were exposed to more than one adverse experience. A Scottish study found that 65% of children experienced more than one ACE^(21)^. Brazilian studies reveal even higher percentages: 74.4%^(14)^ and 89.7%^(22)^. In the Northeastern region of Brazil, it was found that children exposed to three or more events had lower developmental scores compared to non-exposed children^(23)^. This study identified a similar pattern, with frequent coexistence of multiple ACEs in the same child.

Historically, the focus on childhood centered on reducing infant morbidity and mortality, a priority that dominated the past two decades. However, with the advent of the SDGs, this focus has broadened to include economic, environmental, and social determinants aimed at reducing disparities^(7)^. This shift aligns with the findings of this study, highlighting how inequalities generate individual and family vulnerabilities, creating fertile ground for childhood adversities^(24)^. The study’s findings underscore an urgent need to intensify efforts to achieve the SDGs.

Violence during childhood was a prominent finding in this study, manifesting through neglect, abandonment, and other forms within the domestic environment. National and international studies corroborate this reality, indicating that exposure to any type of violence exacerbates developmental challenges, with severe consequences^(22,25,26,27)^. Research shows that children who are victims of violence suffer negative repercussions on their physical and mental health, impacting their adult lives^(5,28,29)^, increasing the likelihood of school dropout^(30)^, and heightening the chances of becoming perpetrators of violence^(31)^, thus compromising their potential to become productive citizens.

Despite the significant findings on violence in this study, it is notable that only 2.2% reported being in a situation of resolution with the Child Protection Council. This may be related to underreporting, as known barriers exist to disclosing domestic violence, even with the widespread implementation of policies for addressing it^(29)^.

Although there are specific SDGs aimed at combating violence against children and adolescents, the literature emphasizes the need for a paradigm shift toward an integrated approach, involving training and capacity-building for professionals across various sectors (health, justice, security, social services) to improve networked collaboration^(27)^. This underscores the urgency of linking the SDGs with children’s rights^(30)^. Health can serve as a point of entry, but cross-sector collaboration is fundamental to reducing disparities and promoting well-being^(1)^.

Health and well-being, as described in SDG 3, have multidimensional social determinants, justifying their interrelationship with other SDGs, such as poverty eradication; zero hunger; quality education; gender equality; clean water and sanitation; decent work and economic growth; reduced inequalities; and peace, justice, and strong institutions^(8)^. All these elements are involved in preventing and addressing ACEs.

In Brazil, achieving the SDG objectives must align with the Unified Health System (SUS), aiming to strengthen intersectorality, universalization, and health equity—essential requirements for addressing the complexity of the Agenda 2030 themes, considering the social, political, economic, cultural, and environmental determinants of health^(32)^. It is well known that poverty increases children’s vulnerability to violence, including child labor, sexual violence, child marriage, trafficking, and recruitment into criminal activities^(33)^.

The presence of conflicts with the law among caregivers of children was identified in the study, which may be explained by the characteristics of the environment in which these individuals are embedded. A study revealed that structural factors such as substance use and abuse, low education levels, socioeconomic marginalization, gender discrimination, and exclusion, along with insufficient and inadequate public policies, are related to crime^(34)^. In Scotland, the deprivation of liberty for parents of children who experienced ACEs occurred in only 0.4%^(21)^, while in Brazil, the rate was 3%^(25)^.

Substance abuse was also observed in this study, consistent with an American study that linked it to the occurrence of ACEs^(13)^. National data indicate that 6.1% of children who experienced ACEs reported parental use of illicit substances^(25)^.

Exposure to violence at home or in the neighborhood is considered a risk factor for the occurrence of ACEs^(13)^. This study found that living in conflict-prone areas negatively affects child development. The conditions of the neighborhood and the occurrence of violent crimes distinctly shape the upbringing of children. Caregivers residing in high-crime neighborhoods clearly describe how these environments threaten their children’s well-being and restrict their educational opportunities compared to those living in low-crime areas^(35)^.

During pregnancy and childhood, the brain grows extremely rapidly, with cerebral volume increasing significantly, reaching 36%, 72%, and 83% of adult volume at 2 to 4 gestational weeks, 1 year, and 2 years of age, respectively. Therefore, it is essential during this stage to establish neural networks and stimulate the development of cognitive, motor, social, and emotional skills, which will be continuously refined throughout adulthood^(36)^.

Scientific evidence suggests an association between family socioeconomic status and the brain and behavioral development of children^(37,38)^. Neuroimaging studies have identified alterations in brain regions, such as reductions in the volume and development of gray matter, the amygdala, and the hippocampus, along with behavioral assessments indicating lower cognitive functions^(39,40)^ in children raised in low socioeconomic environments.

Globally, the situation shows that children are facing extreme poverty and social exclusion more than ever. Before the pandemic, nearly one billion children lived in poverty, and it is now estimated that this number has increased by 10%^(30)^. The situation observed in this study reveals families struggling to acquire food, medicine, and other essential supplies for their children, exacerbated by unemployment and low income, mirroring findings from national and international studies that link economic issues to the occurrence of ACEs^(13,14,21,22,23,24)^.

Lack of infrastructure was also reported by some families, with notable deficiencies in electricity and, especially, basic sanitation. The literature asserts that inadequate social conditions, such as low income, poverty, and extreme poverty, as well as living in precarious environments without sewage systems, potable water, and limited water supply, can exacerbate delays in neurodevelopment^(41,42)^.

Given its significance, SDG Target 6 focuses on access to clean water and sanitation. However, Brazil still shows unsatisfactory performance and requires greater efforts to meet this target by 2030, particularly concerning basic sanitation and access to potable water^(8)^. These objectives should be integrated with other SDGs, such as ending hunger, ensuring quality health, and combating climate change^(43)^.

Strengthening financial security for families and implementing work incentive policies are strategies mentioned in the literature. Decent work promotes sustainable economic growth, enabling families to ensure health, development, and protection for their children. However, it is also necessary to consider social assistance programs, such as cash transfers, to protect childhood^(28)^.

The unfavorable socioeconomic conditions observed among the families included in the study, combined with reports of difficulty in obtaining food, lead to food insecurity, which is reflected in inadequate nutritional conditions due to a diet poor in micro and macronutrients. A study conducted in Brazil associated food insecurity with ACEs. Maternal and child nutrition is essential for brain development and maturation, as well as for maintaining functions throughout life^(36)^.

The expansion of brain size and the support of brain functions require a high demand for energy due to the constant transport of substrates through the blood-brain barrier, necessitating glucose, ketone bodies, fatty acids, and specific nutrients, such as lipids, proteins, and micronutrients^(36)^.

A literature review identified six essential nutrients for maternal nutrition and child brain development: folic acid, iodine, iron, vitamin D, choline, and docosahexaenoic acid (DHA)^(44)^. For children, these nutrients are typically provided through breast milk^(36)^. However, this study demonstrated low adherence to breastfeeding and a high consumption of foods with low nutritional value among the participants. SDG 2, which aims to end hunger, achieve food security, improve nutrition, and promote sustainable agriculture, is a crucial component for achieving many other goals, including safe child development.

Divorce or parental separation was observed in more than half of the studied families, consistent with other research linking it to the occurrence of ACEs^(13,21,22)^. Divorce can lead to changes in family routines, abrupt transitions, altered socioeconomic conditions, reduced contact with one parent, or conflict between parents, all of which can compromise child development^(45)^. When the separation process is contentious, there is an increased risk for the child to develop behavioral disorders, face academic challenges, and engage in substance abuse, with lasting effects into adulthood^(46)^.

A healthy bond with parents is crucial for child development. Studies indicate that children who maintain affectionate relationships with their parents have a reduced risk of developmental issues^(20)^. Programs that support families with children should focus on improving the quality of these bonds, especially for children who experience ACEs.

The health problems of caregivers, as identified in this study, can interfere with the care provided to children, affecting both security and bonding. Similar findings have been reported in other studies, highlighting parental mental health issues^(13,20,21)^ and other diseases^(24)^.

A healthy bond between parents and children protects against childhood adversities; thus, the physical and mental health of parents is essential for ensuring proper care and stimulation for children. Services provided should be family-centered, recognizing the connection between caregiver health and child health^(47)^.

Scientific evidence indicates that prolonged and continuous exposure to ACEs can lead to a condition known as toxic stress^(28,29,48)^, characterized by the prolonged activation of stress response systems in children. This can interfere with the development of brain architecture and other bodily systems, increase the risk of diseases, and cause cognitive issues that persist into adulthood^(48)^. Recognizing adversities such as violence, neglect, poverty, racism, and social isolation is crucial to understanding their potential to trigger toxic stress responses and inhibit the formation of secure, stable, and nurturing relationships^(49)^.

A study conducted on rats demonstrated that early-life stress induces excessive synapse elimination via the astrocyte pathway, permanently remodeling neural circuits and making the animals more prone to developing abnormal behaviors in adulthood^(50)^. Astrocytes and oligodendrocytes play a crucial role in the development and maturation of brain connectivity throughout childhood^(36)^.

Children exposed to toxic stress may exhibit deficits in socio-emotional performance, difficulties in relationships, job instability, lower economic productivity, and depression throughout their lives, which can increase the likelihood of perpetuating intergenerational poverty^(28,30)^.

Given the importance of this topic, sustainable development cannot be achieved if children do not have fair opportunities^(8)^. The 2030 Agenda represents an opportunity to place child protection at the center of political actions for all nations, aiming to build a world where all children enjoy freedom in all its forms^(45)^. This agenda is committed to both the present and the future, striving to promote peaceful and inclusive societies by 2030 through addressing various issues such as ACEs, including racism, and ensuring the provision of quality services, especially in health and education^(8)^.

### Study limitations

One of the study’s limitations relates to participant attrition, which may have led to either an underestimation or overestimation of findings regarding ACEs in the population studied. Additionally, the study design used does not allow for establishing cause-and-effect relationships.

### Contributions to Nursing, Health, or Public Policy

The primary contribution of this study to nursing, health, and public policy lies in the need to disseminate knowledge and raise awareness about the necessary care during early childhood, considering environmental conditions. The study helps strengthen policies aimed at combating risk situations and social vulnerability by providing adequate support for children and their families to reach their full potential. In this context, efforts to achieve the SDGs present an opportunity for more comprehensive action in this area of care.

## CONCLUSIONS

The prevalence of ACEs was 18.6%, as evidenced in the study. Regarding the types of events, income issues, violence, divorce, substance abuse, and local infrastructure problems were the most prevalent. The study also observed the coexistence of ACEs in the same child, highlighting the multifactorial nature of factors affecting childhood, particularly for children already at risk at birth due to biological issues. Unprotected environments, neglect, abandonment, as well as environmental, social, relational, and biological problems in early life represent significant threats to both child and social development.

The coexistence of ACEs in the same child can lead to devastating consequences, with significant impacts on a healthy childhood and implications for academic, health, and social outcomes. It can be concluded, therefore, that meeting the goals of the 2030 Agenda encompasses the dimensions necessary to address this issue by tackling the demands of child development with a multidimensional perspective. Investing in childhood is a crucial catalyst for achieving the SDGs, as it promotes improvements in the socioeconomic and environmental conditions of families, with direct impacts on human development in the short, medium, and long term.
