# Supplementary material for: Adverse childhood experiences among high-risk children living in socially vulnerable areas
Source: Rev Bras Enferm. 2025 Mar 17;78(Suppl 2):e20240247. doi: 10.1590/0034-7167-2024-0247 (PMC11913427; doi:10.1590/0034-7167-2024-0247)
Supplement: Translation

# Translation: Experiências adversas na infância entre crianças de alto risco residentes em locais de vulnerabilidade social

**DOI:** 10.1590/0034-7167-2024-0247pt

**Authors:** Leticia Gramazio Soares, Sabrina dos Santos Tomé, Isabella Schroeder Abreu, Maicon Henrique Lentsck, Tatiane Baratieri, Jorge Marcelo Sauka, Isadora Bussolaro Viana, Kelly Cristina Michalczyszyn

**Affiliations:** I Universidade Estadual do Centro-Oeste. Guarapuava, Paraná, Brasil; II Centro Universitário Campo Real. Guarapuava, Paraná, Brasil

**Keywords:** Experiências Adversas da Infância, Assistência Integral à Saúde, Populações Vulneráveis, Desenvolvimento Infantil, Criança

## Abstract

**Objetivos:**

identificar a ocorrência de experiências adversas na infância entre crianças de alto risco ao nascer.

**Métodos:**

rstudo quantitativo, transversal e descritivo, realizado em um Consórcio Intermunicipal de Saúde no Paraná, de setembro de 2022 a fevereiro de 2023, com 45 cuidadores de crianças de alto risco. A coleta de dados ocorreu no domicílio, utilizando três questionários. Os resultados foram analisados de forma descritiva, com base na teoria da árvore dos eventos adversos da infância.

**Resultados:**

a prevalência de eventos adversos na infância foi de 18,6%. Em relação aos tipos de eventos, 64,3% relataram violência; 28% relataram divórcio; 22,2% relataram abuso de substâncias; 73,3% apresentaram dificuldade para adquirir produtos básicos; 62,2% estavam em situação de desemprego e/ou baixa renda; 55,6% residiam em áreas de conflito; e 44,4% não tinham acesso a esgoto.

**Conclusões:**

os eventos adversos na infância são multicausais e intersetoriais, representando ameaças ao desenvolvimento infantil. A Agenda 2030 propõe dimensões para o enfrentamento dessa problemática, ao investir na infância.

## INTRODUÇÃO

A primeira infância, período que compreende os seis primeiros anos de vida, é uma etapa crucial na vida dos indivíduos, quando se estrutura a arquitetura cerebral, caracterizada pela rápida neuroplasticidade^(1)^, pois o cérebro é moldado a cada experiência vivenciada pela criança^(2)^, momento em que ela adquire a capacidade de aprender^(3)^. A forma como a aprendizagem é estimulada nesse período torna-se o alicerce para o desenvolvimento humano nos anos subsequentes^(4)^.

Quando a criança passa por situações negativas, essas experiências podem causar danos físicos e/ou psicológicos, que podem interferir no desenvolvimento cerebral, desregular o sistema imunológico e neuroendócrino, comprometer a aprendizagem, dificultar a criação de vínculos e impactar as relações sociais a curto, médio e longo prazos. A literatura define essas situações como experiências adversas na infância (EAI)^(4,5)^.

Crianças expostas a EAI, ao chegarem à fase adulta, apresentam maior risco de uso/abuso de substâncias, gravidez indesejada, desenvolvimento de doenças crônicas, aquisição de infecções sexualmente transmissíveis e psicopatologias, além de dificuldades em alcançar seu potencial intelectual, social e econômico^(4,5,6)^.

Com o advento dos Objetivos de Desenvolvimento Sustentável (ODS), o campo da saúde infantil passou a priorizar a melhoria dos resultados no desenvolvimento infantil, além da redução da mortalidade^(1,7)^. Assim, a infância tornou-se uma prioridade internacional, visando oferecer a todas as crianças a oportunidade de atingir seu pleno potencial de desenvolvimento^(5)^, especialmente às menos favorecidas, com o objetivo de garantir um planeta saudável para as gerações presentes e futuras^(8)^.

Desse modo, todos os 193 Estados-membros da Organização das Nações Unidas (ONU), incluindo o Brasil, comprometeram-se a adotar uma agenda e cumprir os 17 ODS, que representam um plano de ação global para melhorar a qualidade de vida da sociedade até 2030^(8)^.

A Rede de Ação para o Desenvolvimento da Primeira Infância, composta pelo Fundo das Nações Unidas para a Infância, Banco Mundial e Organização Mundial da Saúde, propôs um modelo de cuidado que inclui saúde, nutrição, cuidados responsivos, proteção, segurança e aprendizagem desde os primeiros anos de vida^(9)^. No Brasil, o fortalecimento dessa agenda resultou na instituição do Marco Legal da Primeira Infância, que estabelece princípios e diretrizes para políticas públicas nos primeiros anos de vida^(10)^.

Contudo, a classificação de crianças com alto risco ao nascer tem se tornado cada vez mais frequente nos últimos anos, evidenciando um olhar dos serviços de saúde para as experiências negativas que podem interferir no crescimento e desenvolvimento infantil^(11)^. Um estudo sobre a hospitalização nos primeiros anos de vida indica que essa condição pode ter um impacto negativo sobre o crescimento e desenvolvimento, a curto e longo prazos^(12)^.

A vulnerabilidade social é reconhecida pela literatura como uma EAI, pois expõe as crianças às consequências da desigualdade social, incluindo pobreza, exclusão social e falta de acesso à educação, trabalho, saúde, lazer, alimentação e cultura^(13,14)^. Estudos defendem que a melhor maneira de investir na infância é reduzir as desigualdades e construir uma sociedade com condições sustentáveis^(15,16)^.

Evidências provenientes das neurociências indicam uma necessidade urgente de aumentar a cobertura multissetorial na infância, incorporando saúde, nutrição, proteção contra a violência e educação, para que as crianças desenvolvam habilidades necessárias para se tornarem adultos saudáveis e produtivos^(15)^.

Dentro desse contexto, é fundamental promover a conscientização social e estatal sobre as EAI e suas consequências no desenvolvimento humano. Por esse motivo, o tema precisa ser explorado, rastreado e mitigado pelo poder público por meio de estratégias de prevenção e enfrentamento. Esta é uma lacuna identificada em estudos disponíveis na literatura^(17,18)^ e que este estudo busca preencher, trazendo o tema para discussão e divulgação na comunidade científica. É momento de ampliar o debate para auxiliar gestores e profissionais de saúde a valorizar a primeira infância, enxergando-a como uma janela de oportunidades para o desenvolvimento humano. As ações voltadas para a infância devem integrar os ODS às políticas de saúde, visando prevenir e reduzir a exposição às EAI.

## OBJETIVOS

Identificar a ocorrência de EAI entre crianças classificadas como de alto risco ao nascer.

## MÉTODOS

### Aspectos éticos

Este estudo seguiu as orientações da Resolução nº 466/2012 do Conselho Nacional de Saúde e foi aprovado pelo Comitê de Ética em Pesquisa em Seres Humanos. Os participantes da pesquisa preencheram o Termo de Consentimento Livre e Esclarecido (TCLE).

### Desenho, período e local do estudo

Trata-se de um estudo de caráter quantitativo, transversal e descritivo, realizado por meio de visita domiciliar a famílias cujas crianças de alto risco estavam em seguimento no Ambulatório de Pediatria, serviço oferecido pelo Consórcio Intermunicipal de Saúde Guarapuava e Pinhão (CIS-GAP), situado no município de Guarapuava, Paraná, Brasil. O objetivo do serviço é oferecer consultas, exames e procedimentos especializados à população dos municípios consorciados. A escolha por essa instituição deve-se ao fato de ser o serviço que assiste os recém-nascidos classificados como de alto risco, aqueles que apresentam maior chance de evolução desfavorável em razão de mortalidade e morbidade. O estudo foi realizado no período de setembro de 2022 a fevereiro de 2023. Para sua elaboração, foram seguidas as recomendações do *Strengthening the Reporting of Observational Studies in Epidemiology* (STROBE).

### População do estudo; critérios de inclusão e exclusão

A população foi composta pelos cuidadores de crianças classificadas como de alto risco ao nascer, que vivem em áreas de vulnerabilidade social. Na seleção dos participantes, foram adotados os seguintes critérios de inclusão: cuidadores com guarda legal de crianças de alto risco ao nascer, que estavam em acompanhamento no ambulatório do CIS-GAP no período de setembro de 2022 a fevereiro de 2023; crianças na faixa etária de zero a dois anos; residirem em bairros periféricos de Guarapuava, caracterizados por vulnerabilidade social; e que o cuidador fosse maior de idade e tivesse disponibilidade para responder ao questionário. As áreas de vulnerabilidade social incluídas na pesquisa foram aquelas reconhecidas pelo serviço de saúde municipal, caracterizadas por baixa renda, desemprego, problemas de infraestrutura e precariedade ambiental.

Os critérios de exclusão foram: cuidadores sem condições cognitivas para responder; cuidador não encontrado em casa no dia da visita; ou mudança de endereço.

Primeiramente, foi realizado um levantamento do número de crianças de alto risco em atendimento no serviço, resultando em um total de 242 crianças. Após a aplicação dos critérios de inclusão, foram selecionadas 92 crianças para participarem do estudo, das quais 45 efetivamente participaram. As perdas ocorreram pelos seguintes motivos: mudança de endereço (26); cuidador responsável não encontrado em casa (18); recusa em participar (3).

A coleta de dados foi realizada no domicílio das famílias, devido à impossibilidade de realizá-la no serviço em função de uma reestruturação física do local que estava em andamento no período da coleta. Foram aplicados três questionários estruturados: o primeiro contendo dados sociodemográficos; em seguida, um questionário sobre o histórico de saúde da criança, acesso aos serviços, alimentação, crescimento/desenvolvimento e rede de apoio; e, por fim, um questionário contendo questões específicas sobre EAI. Os questionários foram aplicados pelos pesquisadores, previamente treinados e capacitados para essa tarefa.

### Análise dos resultados e estatística

Os resultados foram analisados de forma descritiva, com a apresentação das frequências absolutas e relativas. Para a apresentação dos resultados obtidos na pesquisa, utilizou-se a teoria “*The Pair of Aces*”^(19)^, teoria da árvore dos eventos adversos da infância, que tem como referencial os EAI, representados graficamente em forma de uma árvore, na qual as folhas simbolizam as consequências de crescer em um contexto marcado por pobreza e desigualdades.

## RESULTADOS

Participaram do estudo 45 cuidadores de crianças de alto risco ao nascer, dos quais 17 (37,8%) tinham entre 20 e 25 anos; 29 (64,4%) viviam com companheiros, enquanto 16 (35,5%) não tinham; 29 (64,4%) se declararam brancos e 16 (35,5%), pardos; 29 (64,4%) professavam a religião católica, sete (15,6%) eram evangélicos e nove (20%) declararam não ter religião; 17 (37,8%) tinham ensino médio incompleto, e 11 (24,4%) concluíram o ensino médio; um cuidador (2,2%) iniciou um curso de graduação e dois (4,4%) concluíram o ensino superior. Além disso, um participante não teve acesso à educação formal.

A idade das crianças variou de um a 24 meses, sendo 15 (33,3%) correspondentes à faixa etária de 12 a 18 meses. Quanto à condição que determinou o risco ao nascer, 18 (40%) eram prematuros; 10 (22,2%) apresentavam baixo peso ao nascer; oito (17,8%) tinham malformações congênitas; e nove (20%) apresentavam doenças de transmissão vertical.

Em relação ao aleitamento materno, a única criança menor de seis meses incluída no estudo estava sendo alimentada exclusivamente com leite materno; 55,6% das crianças recebiam, além de leite materno e/ou fórmula, alimentação complementar; destas, 93,3% consumiam frutas e legumes; 91,1% ingeriam verduras; 95,6% consumiam carne; 97,8% comiam arroz, mandioca, batata, farinha e macarrão; 62,2% consumiam embutidos; 66,7% ingeriam bebidas artificiais açucaradas e 71,1% consumiam alimentos industrializados, como biscoitos recheados e salgadinhos.

Oito cuidadores não possuíam a caderneta de saúde da criança, inviabilizando a coleta de informações sobre crescimento e desenvolvimento. No entanto, das 37 cadernetas disponíveis, foi possível verificar as seguintes situações: 22,2% apresentavam atraso no desenvolvimento neuropsicomotor, 66,7% foram diagnosticadas com alguma doença crônica, 28,9% foram internadas no hospital após os 28 dias de vida e 13,3% faziam uso diário de medicação.

Os resultados referentes às EAI levantados nesta pesquisa foram apresentados com base em uma adaptação da imagem originalmente intitulada “*The Pair of Aces*”^(19)^, a qual destaca a importância de ambientes seguros para a infância.

A leitura dos resultados por meio da representação gráfica da árvore permite sistematizar a ocorrência das EAI, organizando os dados em duas classes. A primeira representa situações individuais — as EAI propriamente ditas —, simbolizadas pelos galhos e folhas, e refere-se às adversidades vivenciadas pelas crianças participantes da pesquisa. A segunda classe diz respeito às situações ambientais, representadas pelo solo, denominado de ambiente comunitário adverso (Figura 1).

**Figura 1:** Árvore de Experiências Adversas na Infância

A prevalência de EAI na população geral foi de 18,6%. Quanto à ocorrência e tipos de EAI vivenciados pelas 45 crianças incluídas no estudo, foi possível determinar a seguinte situação: 64,3% das famílias relataram situações de violência, como negligência, desamparo e violência doméstica; em 28,8% das famílias houve divórcio dos pais; em 22,2%, abuso de substâncias por parte dos principais cuidadores da criança; 15,6% dos cuidadores apresentavam algum problema de saúde que interferia no cuidado à criança; e 11,1% dos cuidadores relataram já ter tido conflito com a lei.

Em relação ao ambiente onde as crianças estavam expostas, observou-se uma grande dificuldade para as famílias adquirirem alimentos, medicamentos e demais insumos, sendo relatada por 73,3% das famílias; 62,2% relataram desemprego e baixa renda; 55,6% residiam em áreas de conflito; 44,4% não tinham acesso à rede de esgoto; 2,2% não tinham acesso à rede elétrica; e, por fim, 2,2% estavam resolvendo situações com o Conselho Tutelar.

Os EAI podem envolver diferentes tipos de situações, conforme mencionado. Sobre o número de EAI aos quais cada criança foi exposta, observa-se a seguinte distribuição: 20 crianças (44,5%) foram expostas a dois a cinco eventos; 11 crianças (24,5%) a seis a nove eventos; 10 crianças (22,2%) a apenas um evento; e quatro crianças (8,8%) foram expostas a mais de 10 eventos, o que revela a coexistência de múltiplos eventos adversos em uma mesma criança.

## DISCUSSÃO

Reduzir a exposição às EAI é um desafio mundial, pois questões sociais e estruturais emergem no cenário de estudo e direcionam a discussão para as demandas do desenvolvimento infantil. A literatura indica que há uma relação direta entre a quantidade de EAI e dificuldades no desenvolvimento infantil: quanto maior o número de EAI às quais a criança está exposta, maiores serão as consequências^(20)^. Os resultados deste estudo reforçam essa relação, mostrando que a maioria das crianças foi exposta a mais de uma experiência. Um estudo escocês demonstrou que 65% das crianças vivenciaram mais de uma experiência^(21)^. Estudos brasileiros apontam porcentagens ainda mais elevadas: 74,4%^(14)^ e 89,7%^(22)^. Na região Nordeste do Brasil, foi evidenciado que crianças expostas a três ou mais eventos apresentaram escores de desenvolvimento mais baixos em comparação às crianças não expostas^(23)^. No presente estudo, constatou-se situação semelhante, com a coexistência frequente de EAI na mesma criança.

Anteriormente, a ênfase na infância era centrada na redução da morbidade e mortalidade infantil, foco que prevaleceu nas últimas duas décadas. Contudo, com o advento dos ODS, houve uma ampliação desse enfoque, passando a incluir determinantes econômicos, ambientais e sociais para reduzir as disparidades^(7)^. Essa mudança converge com os resultados deste estudo, ao evidenciar que as desigualdades geram vulnerabilidade individual e familiar, propiciando um terreno fértil para adversidades na infância^(24)^. Os achados do estudo indicam uma necessidade urgente de intensificar os esforços para alcançar os ODS.

A violência na infância se mostrou expressiva neste estudo, manifestando-se por meio de negligência, desamparo e outras formas no ambiente doméstico. Estudos nacionais e internacionais corroboram essa realidade, indicando que a exposição a qualquer tipo de violência é um agravante ao desenvolvimento infantil, com consequências graves^(22,25,26,27)^. Pesquisas demonstram que crianças vítimas de violência apresentam repercussões negativas em sua saúde física e mental que impactam a vida adulta^(5,28,29)^, maior probabilidade de abandono escolar^(30)^ e maiores chances de tornarem-se perpetradoras de violência^(31)^, comprometendo as possibilidades de se tornarem cidadãos produtivos.

Apesar dos resultados expressivos sobre violência no presente estudo, chama a atenção que apenas 2,2% mencionaram estar em situação de resolução junto ao Conselho Tutelar. Esse dado pode estar relacionado à falta de notificação, já que é reconhecido que existem barreiras à divulgação da violência doméstica, mesmo com a implementação generalizada de políticas para enfrentamento^(29)^.

Embora existam ODS que tratam especificamente do enfrentamento da violência contra crianças e adolescentes, a literatura destaca a necessidade de mudar o paradigma para uma abordagem integral, com treinamento e capacitação de profissionais de diversas áreas (saúde, justiça, segurança, serviço social), visando melhor articulação do trabalho em rede^(27)^. Dessa forma, estabelece-se a urgência de interligar os ODS aos direitos da criança^(30)^. A saúde pode ser um ponto de entrada, mas a colaboração entre os setores é fundamental para reduzir as disparidades e promover o bem-estar^(1)^.

A saúde e o bem-estar, conforme descrito no Objetivo 3, possuem determinantes sociais multidimensionais, justificando sua inter-relação com outros ODS, tais como erradicação da pobreza; fome zero; educação de qualidade; igualdade de gênero; água limpa e saneamento; emprego digno e crescimento econômico; redução das desigualdades; e paz, justiça e instituições fortes^(8)^. Todos esses elementos estão envolvidos na prevenção e no enfrentamento das EAI.

No Brasil, o alcance dos objetivos dos ODS deve alinhar-se ao Sistema Único de Saúde (SUS), visando fortalecer a intersetorialidade, universalização e equidade em saúde, requisitos essenciais para abordar a complexidade dos temas da Agenda 2030, considerando os determinantes sociais, políticos, econômicos, culturais e ambientais da saúde^(32)^. É sabido que a pobreza aumenta a vulnerabilidade das crianças à violência, incluindo trabalho infantil, violência sexual, casamento infantil, tráfico e recrutamento para atividades criminosas^(33)^.

A existência de conflito com a lei entre os cuidadores das crianças foi identificada no estudo, o que pode ser explicado pelas características do ambiente em que os sujeitos estão inseridos. Um estudo revelou que fatores estruturais, como uso e abuso de substâncias, baixa escolaridade, marginalização socioeconômica, discriminação de gênero e exclusão, além de políticas públicas insuficientes e inadequadas, estão relacionadas ao crime^(34)^. Na Escócia, a privação de liberdade de pais de crianças que vivenciaram EAI ocorreu em apenas 0,4%^(21)^, enquanto no Brasil foi observado um índice de 3%^(25)^.

O abuso de substâncias foi verificado neste estudo, assim como em um estudo americano que o associou à ocorrência de EAI^(13)^. Dados nacionais apontam que 6,1% das crianças que passaram por EAI relataram uso de substâncias ilícitas pelos pais^(25)^.

A exposição à violência em casa ou na vizinhança é considerada um fator de risco para a ocorrência de EAI^(13)^. Neste estudo, foi constatado que viver em áreas de conflito influencia negativamente o desenvolvimento infantil. As condições da vizinhança e a ocorrência de crimes violentos moldam de forma distinta a criação dos filhos. Cuidadores que residem em bairros com elevada criminalidade descrevem com maior clareza como esses locais ameaçam o bem-estar de seus filhos e restringem sua educação, em comparação com aqueles que vivem em áreas de baixa criminalidade^(35)^.

Durante a gravidez e a infância, o cérebro cresce de forma extremamente rápida, com o volume cerebral aumentando significativamente, atingindo 36%, 72% e 83% do volume de um adulto, nas 2ª a 4ª semanas gestacionais, aos 1 anos e 2 anos de idade, respectivamente. Portanto, é essencial, nessa fase, estabelecer as redes neuronais e estimular a capacidade para o desenvolvimento de habilidades cognitivas, motoras, sociais e emocionais, que serão continuamente refinadas ao longo da vida adulta^(36)^.

Evidências científicas sugerem uma associação entre o nível socioeconômico das famílias e o desenvolvimento cerebral e comportamental das crianças^(37,38)^. Estudos de neuroimagem já identificaram alterações em regiões cerebrais, como redução no volume e desenvolvimento da substância cinzenta, da amígdala e do hipocampo, além de avaliações comportamentais que apontam funções cognitivas inferiores^(39,40)^ em crianças que se desenvolvem em ambientes de baixo nível socioeconômico.

O cenário mundial mostra que, mais do que nunca, crianças enfrentam pobreza extrema e exclusão social. Antes da pandemia, quase um bilhão de crianças viviam na pobreza, mas atualmente estima-se que esse número seja 10% superior^(30)^. A situação verificada neste estudo revela famílias com dificuldade para adquirir alimentos, medicamentos e outros insumos essenciais para as crianças, agravada pelo desemprego e pela baixa renda, de forma semelhante ao cenário demonstrado em estudos nacionais e internacionais, nos quais as questões econômicas foram associadas à ocorrência de EAI^(13,14,21,22,23,24)^.

A falta de infraestrutura também foi relatada por algumas famílias, destacando-se a ausência de luz e, especialmente, de saneamento básico. A literatura afirma que condições sociais inadequadas, como baixa renda, pobreza e extrema pobreza, bem como residências em ambientes precários, sem esgoto, água potável e abastecimento de água reduzido, podem potencializar atrasos no neurodesenvolvimento^(41,42)^.

Dada essa importância, a Meta 6 dos ODS aborda o acesso à água limpa e ao saneamento. No entanto, o Brasil ainda apresenta um desempenho insatisfatório e necessita de maiores esforços para cumprir essa meta até 2030, especialmente em relação ao saneamento básico e ao acesso à água potável^(8)^. Esses objetivos devem ser integrados a outros ODS, como erradicação da fome, saúde de qualidade e combate às mudanças climáticas^(43)^.

Fortalecer a segurança financeira das famílias e implementar políticas de incentivo ao trabalho são estratégias mencionadas na literatura. O trabalho digno promove o crescimento econômico sustentável, permitindo que as famílias garantam saúde, desenvolvimento e proteção aos seus filhos. No entanto, também é necessário considerar programas de assistência social, como transferências de renda, para proteger a infância^(28)^.

O cenário socioeconômico desfavorável observado nas famílias incluídas no estudo, somado ao relato de dificuldade em obter alimentos, leva à insegurança alimentar, refletindo-se em condições nutricionais inadequadas devido a uma dieta pobre em micro e macronutrientes. Um estudo realizado no Brasil associou a insegurança alimentar às EAI. A nutrição materna e infantil é essencial para o desenvolvimento e a maturação cerebral, além de manter funções ao longo da vida^(36)^.

A expansão do tamanho cerebral e a sustentação das funções cerebrais requerem alta demanda de energia, devido ao constante transporte de substratos através da barreira hematoencefálica, necessitando de glicose, corpos cetônicos, ácidos graxos e nutrientes específicos, como lipídios, proteínas e micronutrientes^(36)^.

Uma revisão da literatura revelou seis nutrientes indispensáveis para a nutrição materna e o desenvolvimento cerebral dos filhos: ácido fólico, iodo, ferro, vitamina D, colina e ácido docosa-hexaenoico^(44)^. Em relação às crianças, esses nutrientes são normalmente fornecidos pelo leite materno^(36)^. No entanto, este estudo demonstrou uma baixa adesão ao aleitamento materno e um elevado consumo de alimentos com baixo valor nutricional entre o público. O ODS 2, que visa acabar com a fome, alcançar a segurança alimentar, melhorar a nutrição e promover a agricultura sustentável, é um componente essencial para alcançar muitas das outras metas, como o desenvolvimento infantil seguro.

O divórcio ou a separação dos pais foi verificado em mais da metade das famílias estudadas, assim como em outras pesquisas que o associaram à ocorrência de EAI^(13,21,22)^. O divórcio pode provocar mudanças na rotina familiar, transições abruptas, alterações nas condições socioeconômicas, menor contato com um dos pais ou conflitos entre eles, o que pode comprometer o desenvolvimento infantil^(45)^. Quando o processo de separação é conflituoso, há um risco aumentado de a criança desenvolver distúrbios comportamentais, dificuldades escolares e abuso de substâncias, além de consequências na vida adulta^(46)^.

O vínculo saudável com os pais é crucial para o desenvolvimento infantil. Estudos indicam que crianças que mantiveram relações afetuosas com seus pais apresentam um risco reduzido de problemas no desenvolvimento^(20)^. Programas que atendem famílias com crianças devem concentrar esforços em melhorar a qualidade desses vínculos, especialmente para crianças que vivenciam EAI.

Os problemas de saúde dos cuidadores, verificados neste estudo, podem interferir no cuidado prestado às crianças, afetando a segurança e o vínculo. Resultados semelhantes foram encontrados em outros estudos, que apontam problemas de saúde mental dos pais^(13,20,21)^ e outras doenças^(24)^.

O vínculo saudável entre pais e filhos protege as crianças contra adversidades na infância; portanto, a saúde física e mental dos pais é essencial para garantir o cuidado e o estímulo necessários às crianças. Os serviços oferecidos devem ser centrados na família, reconhecendo a conexão entre a saúde dos cuidadores e a saúde das crianças^(47)^.

Evidências científicas indicam que a exposição prolongada e contínua a EAI pode resultar em uma condição chamada estresse tóxico^(28,29,48)^, caracterizado pela ativação prolongada dos sistemas de resposta ao estresse em crianças. Isso pode interferir no desenvolvimento da arquitetura cerebral e de outros sistemas orgânicos, aumentar o risco de doenças e causar problemas cognitivos que perduram até a idade adulta^(48)^. Reconhecer adversidades como violência, negligência, pobreza, racismo e isolamento social é entender o potencial que elas têm para desencadear respostas de estresse tóxico e inibir a formação de relações seguras, estáveis e estimulantes^(49)^.

Um estudo realizado com ratos demonstrou que o estresse no início da vida induz à eliminação excessiva de sinapses através da via dos astrócitos, remodelando permanentemente os circuitos neurais e tornando os animais mais propensos a desenvolver comportamentos anormais na idade adulta^(50)^. Os astrócitos e os oligodendrócitos desempenham um papel crucial no desenvolvimento e na maturação da conectividade cerebral ao longo da infância^(36)^.

Crianças expostas ao estresse tóxico podem apresentar déficits no desempenho socioemocional, dificuldades em relacionamentos, instabilidade no trabalho, menor produtividade econômica e depressão ao longo da vida, o que pode aumentar a probabilidade de perpetuação da pobreza intergeracional^(28,30)^.

Dada a importância do tema, o desenvolvimento sustentável não será alcançado se as crianças não tiverem oportunidades justas^(8)^. A Agenda 2030 apresenta-se como uma oportunidade para colocar a proteção das crianças no centro das ações políticas de todas as nações, com o objetivo de construir um mundo onde todas as crianças desfrutem de liberdade em todas as suas formas^(45)^. É uma agenda comprometida com o presente e o futuro, buscando promover sociedades pacíficas e inclusivas até 2030, por meio do enfrentamento de diversas situações, como as EAI, incluindo também o racismo e a oferta de serviços de qualidade, especialmente em saúde e educação^(8)^.

### Limitações do estudo

Uma das limitações do estudo está relacionada às perdas de participantes, o que pode ter sub ou superestimado os achados sobre as EAI na população pesquisada. Além disso, o tipo de estudo utilizado não permite estabelecer relações de causa e efeito.

### Contribuições para a área da enfermagem, saúde ou política pública

A contribuição principal deste estudo para a enfermagem, saúde e políticas públicas reside na necessidade de divulgar a temática e alertar sobre os cuidados necessários na primeira infância, considerando as condições ambientais. O estudo contribui para fortalecer políticas que combatam situações de risco e vulnerabilidade social, oferecendo suporte adequado para que as crianças e suas famílias possam alcançar seu pleno potencial. Nesse contexto, o trabalho para alcançar os ODS representa uma oportunidade para atuar de forma mais abrangente nesse cenário assistencial.

## CONCLUSÕES

A prevalência de EAI foi de 18,6%, sendo uma realidade evidenciada no estudo. Em relação aos tipos de eventos, verificou-se que renda, violência, divórcio, abuso de substâncias e problemas de infraestrutura local foram os mais prevalentes. Observou-se também a coexistência de EAI na mesma criança, o que evidencia a natureza multicausal dos fatores que afetam a infância, especialmente em crianças que já possuem um risco ao nascer, relacionado a questões biológicas. Espaços desprotegidos, negligência, desamparo, além de problemas ambientais, sociais, relacionais e biológicos nos primeiros anos de vida representam ameaças importantes ao desenvolvimento infantil e social.

A coexistência de EAI na mesma criança pode gerar prejuízos devastadores, com impactos significativos para uma infância saudável e consequências nos resultados acadêmicos, de saúde e sociais.

Conclui-se, portanto, que o cumprimento da Agenda 2030 abrange as dimensões necessárias para enfrentar essa problemática, ao abordar as demandas do desenvolvimento infantil com uma perspectiva multidimensional. Investir na infância é um importante catalisador para alcançar os ODS, ao promover melhorias nas condições socioeconômicas e ambientais das famílias, com impacto direto a curto, médio e longo prazo no desenvolvimento humano.
